# Sort-seq under the hood: implications of design choices on large-scale characterization of sequence-function relations

**DOI:** 10.1186/s12864-016-2533-5

**Published:** 2016-03-09

**Authors:** Neil Peterman, Erel Levine

**Affiliations:** Department of Physics and FAS Center for Systems Biology, Harvard University, 17 Oxford St., Cambridge, MA USA

**Keywords:** Sequence-function relations, Systems biology, High-throughput sequencing, Fluorescence-activated cell sorting

## Abstract

**Background:**

Sort-seq is an effective approach for simultaneous activity measurements in a large-scale library, combining flow cytometry, deep sequencing, and statistical inference. Such assays enable the characterization of functional landscapes at unprecedented scale for a wide-reaching array of biological molecules and functionalities in vivo. Applications of sort-seq range from footprinting to establishing quantitative models of biological systems and rational design of synthetic genetic elements. Nearly as diverse are implementations of this technique, reflecting key design choices with extensive impact on the scope and accuracy the results. Yet how to make these choices remains unclear. Here we investigate the effects of alternative sort-seq designs and inference methods on the information output using mathematical formulation and simulations.

**Results:**

We identify key intrinsic properties of any system of interest with practical implications for sort-seq assays, depending on the experimental goals. The fluorescence range and cell-to-cell variability specify the number of sorted populations needed for quantitative measurements that are precise and unbiased. These factors also indicate cases where an enrichment-based approach that uses a single sorted population can offer satisfactory results. These predications of our model are corroborated using re-analysis of published data. We explore implications of these results for quantitative modeling and library design.

**Conclusions:**

Sort-seq assays can be streamlined by reducing the number of sorted populations, saving considerable resources. Simple preliminary experiments can guide optimal experiment design, minimizing cost while maintaining the maximal information output and avoiding latent biases. These insights can facilitate future applications of this highly adaptable technique.

**Electronic supplementary material:**

The online version of this article (doi:10.1186/s12864-016-2533-5) contains supplementary material, which is available to authorized users.

## Background

The relation between sequence and function is a central focus of molecular biology. High-throughput techniques have enabled researchers to explore these relations at previously inaccessible scales [1–3]. Methods based on fluorescence-activated cell sorting (FACS) followed by high-throughput sequencing — collectively referred to hereafter as *sort-seq* — allow the measurement of fluorescent reporters in many thousands of genetic variants at high precision in a single experiment [4–9]. Sort-seq offers a window to examine a broad array of processes in vivo with quantitative precision, including in particular aspects of transcriptional and post-transcriptional regulation. Along with other techniques that measure fitness [10–14], protein or ribozyme activity [15–20] or mRNA abundance [21–27] on a massive scale, sort-seq redefines what is possible for studies of sequence-function relations and epistasis.

**Fig. 1:**
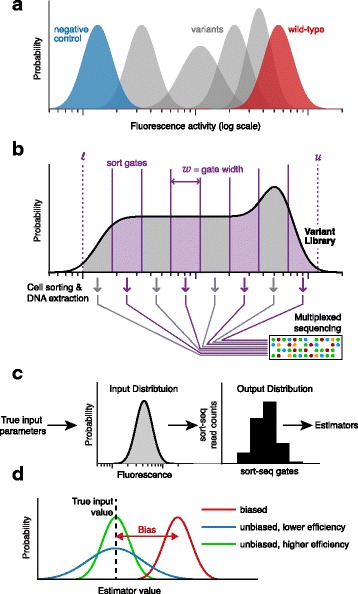
Sort-seq scheme. **a** Input distributions of single-cell fluorescence measurements for individual isolated variants, plotted as histograms. Distributions for these variants may have different mean and variance from the wild-type or reference variant. **b** The distribution is much broader for the mixed library of variants. Cells are sorted by flow cytometry and sequenced in parallel. Sort gates define each sorted population and span the fluorescence range between *ℓ* and *u*. In this configuration, gates are evenly spaced with width *w* on a log scale. **c** The input distribution of single-cell fluorescence measurements for a single variant is characterized by input parameters. The output distribution represents the proportions of sort-seq reads within each gate. Statistical estimators are used to infer the input parameters from the output distribution. **d** The performance of an estimator is characterized by examining the probability that it yields a certain value in an experiment (colored curve) compared with the true input value (indicated in black). For a biased estimator (red) this probability distribution is not centered around the true input parameter, and one defines the bias as the distance between its mean and the true value. Unbiased estimators (blue, green) are centered around the input. The efficiency of such estimators depends on the width of the distribution, such that with a given sample, a more efficient estimator (green) leads to a more precise estimate

A typical sort-seq experiment (Fig. 1a–b) begins with a mixed population or library of variants of a given gene or sequence of interest, whose function is indicated by a fluorescent reporter. Cells are sorted and binned according to flow cytometry measurements such as fluorescence in one or more channels. Sorted subpopulations are then sequenced in order to parse the distribution of different variants across the sorting bins. These data may then be used to infer the activity of each variant, allowing the direct characterization of sequence-function relations on a large scale.

This high-throughput technique has been proven useful in deeply characterizing sequence-function relations in transcriptional regulation [4, 5,8, 28–30], 5’ or 3’UTRs of mRNAs [7,31–34], regulatory RNAs [9, 35], and a variety of other systems [36–39]. Sort-seq has been demonstrated in bacteria [4,6,9,35,36,38], yeast [5,8, 31, 37] and mammalian cells [7,29, 30, 32,40], as well as tissues from multicellular organisms [41]. Among these experiments there are subtle but important differences in how sort-seq is performed. Some use a single gate, which defines the range of fluorescence measurements for cells to be sorted, and then measure enrichment relative to an unsorted population [29, 30, 35–37]. Others employ multiple gates to quantify fluorescence [4–7,9, 31, 32, 38], using as few as four [9] and as many as 32 [8]. The number of sort events or reads per variant ranges from one [4] to thousands [6, 36]. Some use an additional constitutive reporter in a different color channel [5,6, 8, 31,38], while others do not. The library of variants itself can be based on random mutations with higher [4] or lower frequency [9], or on more fine tuned randomization schemes [5, 6, 36, 38, 42–44]. These choices represent experimental trade-offs, often between the cost and complexity of the assay on one hand and the scope and quantitative precision of the measurements on the other.

A robust and efficient design of a sort-seq experiment therefore requires an understanding of how the diverse design choices impact the scale and fidelity of its output. Here, we use a combination of modeling, simulations and reanalysis of published datasets to characterize the information output of different sort-seq experimental configurations and analysis methods. We show how principal qualities of these outputs depend on the way key properties of the system under investigation are related to specific aspects of the sort-seq procedure. An optimal design of a sort-seq experiment therefore relies on correctly estimating these properties, and on matching the quality of the expected results with what is needed to achieve the goals of the experiment. We use our results to suggest a possible workflow for designing, executing, and analyzing a sort-seq experiment.

## Results and discussion

### Defining the problem: inferring the mean and dispersion of mixed input distributions

The assumption behind a sort-seq experiment is that each variant of the investigated sequence can be associated with a single quantity that represents it activity. This can be, for example, the equilibrium affinity of an enzyme to its substrate, the transcription rate from a promoter, the modulation of target expression by a small regulatory RNA, etc. The activity is represented in the cell via a fluorescent reporter.

Due to cell-to-cell variability and experimental noise, a population of cells that carry the same variant yields a distribution of fluorescence measurements. This distribution is referred to below as the *input distribution* (Fig. 1c). The quantity of interest can be represented as some attribute of the input distribution, e.g. in the simplest case its mean. The biological contributions to the shape of this distribution can carry information about the structure of the population or about the system’s mechanisms of action. Thus in some cases one is interested in estimating other properties of the input distribution, such as its dispersion (that is, how much it is stretched or squeezed).

Sort-seq methods aim to infer the activity (and perhaps its cell-to-cell variability) for a large number of sequence variants in parallel, by estimating the statistical attributes of each input distribution in the mixed population (the *library*). For each variant, sort-seq provides the distribution of each variant among the different sorting gates (the *output distribution*), which can be thought of as a low-resolution sampling from the input distribution (Fig. 1c). The low resolution comes from the fact that all we know about the fluorescence of each cell is that its measurement lies somewhere within the corresponding gate, but we do not know the measurement value itself. The task is to infer from these data the relevant attributes of the input distribution. For concreteness, we focus here on estimating the mean of each input distribution and its dispersion, quantified by the coefficient of variation (CV, the ratio between the standard deviation and the mean).

### Parameterizing the sort-seq configuration and the quality of its results

The configuration of a sort-seq experiment is defined by the choice of sorting gates. As an approach that can cover a very wide dynamic range, one can use fluorescence intervals evenly spaced on a log scale (*log-spaced gates*). A configuration is then specified by the number of gates *m* and the range of gated measurements, defined by the measurement boundaries *u* and *ℓ*. The width of each gate is then given by *w*=*log*(*u*/*ℓ*)/*m* (Fig. 1b). Choosing a larger number of gates means higher cost and greater effort, but can potentially improve the resolution of the sort-seq assay.

To gauge the success of a sort-seq design, we asses the quality of the estimates it produces. Two desirable properties of estimators are their accuracy and efficiency [45]. The accuracy of an estimator is quantified through its bias, defined as the mean error of the estimator with respect to the input parameter it estimates. For an unbiased estimator (that is, an estimator whose bias is zero), averaging the estimates obtained from very many repeats of the experiment yields the estimated quantity exactly (Fig. 1d).

Another property of an estimator is its mean square error (MSE), which measures how far an estimate is expected to be from the true value it aims to estimate. For an unbiased estimator, the MSE also quantifies the precision of the experiment, that is how far the estimates from different repeats are expected to be from one another. When the MSE is large, the estimate from a single experiment can be very different from the true value, even if the estimator is unbiased.

Efficiency provides a measure for the optimality of the experimental design, such that a more efficient estimator requires fewer measurements to yield a given level of precision (Fig. 1d). To quantify the efficiency of an estimator, we take the ratio between the MSE that results from estimating the parameter in a standard flow cytometry experiment with a given number of cells, and the MSE of the estimator in a sort-seq experiment with the same number of cells per variant. The former is equivalent to a sample taken from the true input distribution, and can be thought of as a limit of how well one can do with a sample of this size. Intuitively, when the estimator is unbiased, the efficiency is just the ratio between the number of individual flow cytometry measurements and the number of sort-seq measurements required to achieve the same precision.

### Gate and reporter configurations for accurate and efficient quantification of single-cell fluorescence

In order to evaluate the quality of the expected outcome for different sort-seq configurations, we simulated the results of sort-seq experiments. In each simulation, we assume a population of sequence variants whose mean fluorescence covers the entire range. Each sequence variant was defined by an input distribution with a different mean and a fixed CV. These distributions are taken to be log-normal, a minimal distribution that well-approximates flow cytometry measurements in bacteria and yeast with diverse reporters and cellular conditions [46].

We represented every sequence variant in the sample by *N*=100 different cells, the fluorescence value of which was drawn randomly from the input distribution of that variant. These cells were then placed in the appropriate sorting gate based on the sort-seq configuration under investigation. Every variant is therefore assigned an output distribution, namely the way the *N* cells are distributed among the *m* sorting gates. These data are then used to estimate the input mean of that variant, as explained below. We repeated the simulations many times in order to quantify the accuracy and efficiency of the estimator for each value of the input mean. Details of our numerical procedures can be found in “Methods: Sort-seq simulations”.

**Fig. 2:**
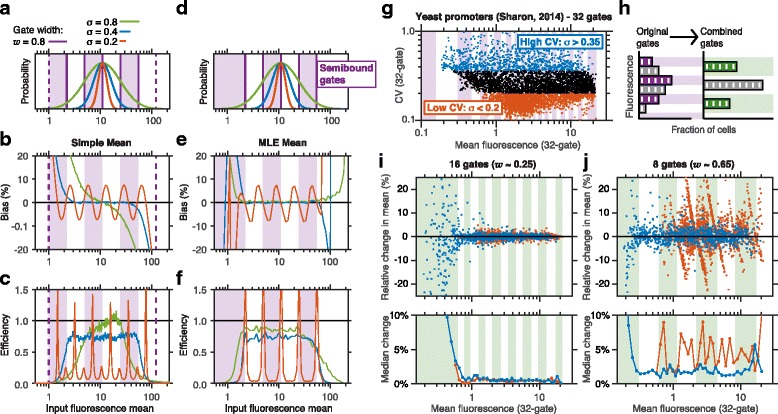
Bias and efficiency of mean fluorescence estimates. **a** Histograms of input fluorescence distributions for three variants with different cell-to-cell variability. Shaded regions indicate the 6 log-spaced sort gates (*w*=0.8). Dashed lines bound the two outer gates. **b** Relative bias and **c** efficiency of the simple mean estimator from sort-seq simulations, plotted against the input mean for variants with different levels of *σ*. **d** Gate configuration for MLEs, which feature semibound gates that capture all cells with fluorescence above or below thresholds on the right and left, respectively. **e** Relative bias and **f** efficiency plotted for the MLE mean as in **b**–**c**. Simulations used *N*=100 sort-seq reads per repeat, averaging over 1000 repeats per set of parameter values. (G) A sort-seq dataset [8] is used to infer mean and CV using MLEs for 5255 yeast promoters. Inferred CV is used to define variants with high CV (*σ*>0.35, blue) and low CV (*σ*<0.20, red). Shaded regions indicate original sort gates. **h** Sort-seq gates were re-grouped by combining reads corresponding to adjacent gates, resulting in larger gate width *w*. **i**–**j** Relative change between the estimates of the mean using the re-grouped data and the full data. **i** 16 gates (*w*≈0.25) and **j** 8 gates, (*w*≈0.65). Lower panels indicate median absolute change. Shades indicate re-grouped gates

There are many ways in which one can use the output distribution of a variant to estimate the attributes of the input. We begin by considering a rudimentary estimator of mean fluorescence (hereafter the *simple mean*), where all reads in a given sorting gate are assigned a fixed value in that gate, and are averaged for each variant. (see Methods: Quantitative estimation with sort-seq). This or a similar approach has been utilized in a number of sort-seq studies [5, 6, 8, 34]. We explored the bias and efficiency of the simple mean estimator by simulating sort-seq sampling for variants with a range of mean fluorescence values and with different levels of cell-to-cell variability. This was done with 6 log-spaced gates and *w*=0.8 (Fig. 2a–c), as well as with several other configurations (Additional file 1: Figure S1).

The simple mean was found to be both unbiased and efficient for variants with *σ*>*w*, as long as the estimated mean is not too close to the measurement boundaries (e.g. Additional file 1: Figure S1D,G, red lines, with additional values of *σ* plotted in the heatmaps of this figure). On the other hand, for variants with *σ*<*w*/2 the simple mean can be biased and sensitive to small changes in fluorescence (e.g. Fig. 2b, red), introducing systematic errors that are strongly dependent on the sorting configuration. Intuitively, this is because variants with such narrow input distributions are not spread enough among the sorting gates to allow accurate estimates. For variants with intermediate cell-to-cell variability (*σ*≥*w*/2 but smaller than *w*, e.g. Fig. 2b, c, blue, and Additional file 1: Figure S1D,G, amber) the simple mean estimator remains unbiased, however somewhat less efficient. Finally, regardless of gate width, the simple mean is highly biased for variants whose mean is within a factor of *e*^2*σ*^ of the measurement boundaries (Fig. 2b, Additional file 1: Figure S1D–F), reflecting a sensitivity of this estimate to even a small fraction of missing measurements. In sum, the simple mean works very well for variants whose input distribution is of the same order as the gate width or larger, provided that their input mean is not too close to the measurement boundaries.

An alternative to the simple mean is a maximum likelihood estimator (MLE), which has also been used to quantify fluorescence using sort-seq data [9]. Here one uses some assumptions or external information about the shape of the input distributions. This added information has the potential to improve performance, especially for variants where some of the data is missing (e.g., variants for which some of the cells fall outside the measurement range). In contrast to the simple estimators, MLEs support the use of semibounded gates, namely gates that have either an upper bound or a lower bound, but not both (respectively the leftmost and rightmost gates in Fig. 2d). These gates ensure that cells at all fluorescence levels are captured and accounted for. The success of an MLE approach requires a reliable assumption about the shape of the input distribution; here we assume that this distribution is log-normal, as is often the case in measurements of gene expression [46].

In order to compare these two estimators, we assessed the performance of MLEs using the same number of gates and gate-width as above (Fig. 2d–f, Additional file 1: Figure S2A–C). The MLE approach has similar performance in estimating the mean as the previous approach, with one significant advantage: a substantial reduction in the bias near the measurement boundaries (cf. Fig. 2b, e, green). Thus MLEs can expand the range of inputs for which reliable quantitative measurements can be obtained.

As mentioned above, noise in gene expression, namely cell-to-cell variability in the concentration of a particular protein, is an important property of genetic control elements [47]. For a population of cells, this variability can be used, for example, to increase survival in an unpredictable environment [48–50]. Properties of the noise can also carry information about the system’s mechanisms of action [50, 51]. Thus, sort-seq experiments may be tasked with estimating the strength of the noise for each variant through its estimated dispersion (that is, the coefficient of variation CV), as was done in [8]. Importantly, the inferred CV is influenced not only by this biologically relevant noise, but also by other factors, such as measurement noise and sorting errors. With an appropriate model for these different factors, one can in principle separate the different sources of noise a posteriori [52].

**Fig. 3:**
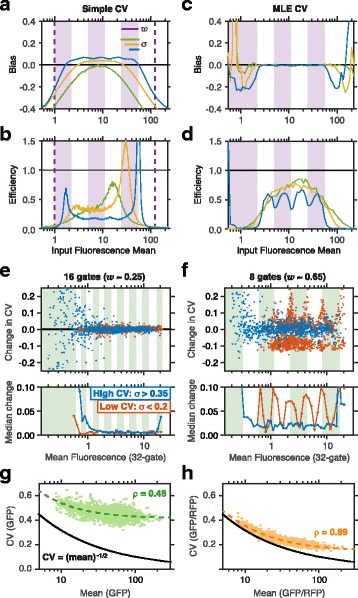
Estimates of cell-to-cell variability using sort-seq. **a**–**d** Sort-seq simulations using the same configurations as in Fig. 2 a–f. Bias and efficiency of **a**–**b** the simple CV and **c**–**d** the MLE CV were plotted as in Fig. 2. **e**–**f** The effect of the number of gates on CV estimates from the yeast promoter dataset [8]. The change between the CV estimate using data from combined gates and the full dataset is plotted for two combined gate configurations (the same as in Fig. 2 i–j) for variants with different levels of CV, as estimated from the original data. Lower panels indicate median absolute change. **g**–**h** Sort-seq estimates of mean and CV from simulations using **g** a single reporter (GFP) and **h** two reporters (GFP and a reference reporter RFP). Spearman’s correlation *ρ*=0.48 for GFP **g**, and *ρ*=0.89 for GFP/RFP **h**. Dashed lines indicate the relationship between input mean and CV in each system, and the solid line is the prescribed relationship between mean and the intrinsic component of cell-to-cell variability

The CV can be inferred using estimators analogous to the ones described above, the simple CV and the MLE CV (see “Methods: Quantitative estimation with sort-seq”). Simulations reveal that the simple CV tends to overestimate the noise for variants with *σ*<*w*, and to underestimate it for variants whose mean is near the measurement boundaries or when *σ*>*w* (Fig. 3a–b, Additional file 1: Figure S3). Conversely, the MLE CV performs nearly as well as the MLE of the mean (Fig. 3c–d, Additional file 1: Figure S2D-E): it is unbiased when the input mean is within the sorted range and *σ*≥*w*/2, and has nearly optimal efficiency for *σ*≥*w*. Together, we conclude that with a maximum-likelihood approach, the sort-seq assay can make accurate estimates for all variants with *σ*≥*w*/2.

We sought to verify these simulation results by considering the data from a recent sort-seq experiment, which aimed to probe the effect of promoter sequence on gene expression in yeast [8]. In this study, both expression level and cell-to-cell variability were characterized for several thousand promoters using 32 sorting gates (Fig. 2g, Additional file 1: Figure S4). These data can be used to study how the number and width of sorting gates can affect the output of the experiment by combining reads from adjacent gates in the original experiment to generate the expected output from experiments with other gate configurations (Fig. 2h, and see Methods: Reanalysis of sort-seq data). We then used these data to estimate the mean and CV using MLEs. We assumed that the estimate of mean fluorescence obtained in the original experiment yields the correct input value (the “ground truth”), and estimated the relative error between this value and the one obtained from each different gate configuration (Fig. 2i–j, Additional file 1: Figures S5 and S6).

Variants in the yeast promoter library differed substantially in both mean and CV [8], allowing us to test our prediction that reliable estimates can only be achieved for variants whose CV is larger than half the width of the gates. To do this, we divided the range of CV estimates into three roughly equal parts (in log-scale, Fig. 2g), and focused on variants with high CV (blue, *σ*>0.35, 17 % of variants) and low CV (*σ*<0.20, 39 % of variants).

Reducing the number of gates from 32 in the original study to 16 (corresponding to *w*≈0.25) had little effect on the MLE mean for variants with a broad range of mean fluorescence levels (Fig. 2i), irrespective of their CV. This is consistent with the predictions of our model, as in this case *σ*≥*w*/2≈0.12 for nearly all variants. However, when gate width was further increased (*w*≈0.65, 8 gates), estimates for many variants became significantly biased (Fig. 2j). As predicted from our model (Fig. 2e red), this bias was significant for variants with small CV (Fig. 2j, red), as for these variants *σ*<*w*/2, but remained small for variants with high noise (compare data in blue in Fig. 2j and blue curves in Fig. 2e), for which *σ*≥*w*/2. Reduction in the number of gates had a similar effect on estimates of CV for each variant (Fig. 3e–f).

Overall we conclude that a significant fraction of the measurements in this assay – but not all – could tolerate a substantial reduction in the number of gates with minimal effect on their accuracy. As expected from our model analysis, the variants most sensitive to re-binning are those with low CV and those near the measurement boundaries. The choice of gate width, and thus the range of variants that could be estimated reliably, should be guided by the goals of the experiment.

A significant contribution to noise in gene expression comes from extrinsic factors, such as cell size or DNA copy number [47]. One way to control for these factors is to co-express a variant reporter and a reference reporter in each cell, and measure the ratio between the two [5, 6, 8]. To evaluate the benefit of this approach we simulated sort-seq measurements for variants whose cell-to-cell variability have a constant extrinsic component as well as an intrinsic component that scales as the square root of the mean (see Methods: Dual-reporter simulations). We supposed that a researcher is interested in recovering this relationship between mean and variance in gene expression, and attempts to gauge her success when following a sort-seq procedure with or without the reference reporter. By plotting the estimated CV against the estimated mean for each variant (Fig. 3g–h), we found that an added reference reporter significantly aids the ability to detect the embedded relation between the intrinsic noise and the mean (Spearman’s correlation *ρ*=0.48 without the added reporter, Fig. 3g, and *ρ*=0.89 with it, Fig. 3h). This improvement reflects a closer estimate of the underlying intrinsic noise (compare dashed and solid lines) as well as a significant increase in precision (that is, reduction in the scatter of the estimated data points around the dashed line).

In summary, the choice of gate configuration has a strong effect on the quality of sort-seq estimates, in particular for variants whose input distributions are narrower than the width of the sorting gates or in cases where a significant fraction of fluorescence measurements fall outside the measurement boundaries. The gate configuration should therefore be designed such that the width of the gates is comparable to the width of a typical input distribution, and such that variants of interest are mostly within the measurement boundaries. Use of a larger number of gates, which may be costly in time, labor, and biological material, is expected to yield only a marginal improvement in the results. A reference reporter, which allows separating different sources of noise, can help in characterizing noise properties of the system under study. This may require a corresponding increase in the number of sorting gates, but can also improve the precision of the results.

### Fitting quantitative models using sort-seq data

While sort-seq can yield precise measurements for a large number of variants, one can only ever expect to measure a small subset of all possible variants since their number expands rapidly as the number of mutations increases. One can examine the measured effect of each individual mutation or even pairs, however this describes just one corner of a deep and complex landscape. Instead we aim to extrapolate the data by establishing a quantitative model describe and understand these landscapes, which can be inferred using sort-seq data from a diverse mutant library [4–9, 31, 38, 53, 54]. Parameters of this model may correspond to biochemical properties such as reaction rates or molecular structures, coarse-grained features, and more.

Given a model for how sequence is linked with activity, the task is to infer the parameters of the model from sort-seq data. In some cases there is a clear relationship between the activity of the system under study and mean fluorescence, for example the fold-change in fluorescence caused by expression of a regulatory RNA gene. In such cases, the parameters of the model can be inferred directly from the estimates of the mean. In other cases, the activity affects the level of reporter fluorescence in a complex (though typically monotonic) way. For example, the activity of interest could be the affinity of an enzyme to a substrate, while the fluorescence reports the rate of the reaction. If a good model for mapping activity to fluorescence exists (such as the Michaelis-Menten model in the last example), one can still use a maximum likelihood approach to infer all parameters of the combined model simultaneously, and also estimate estimate cell-to-cell variability.

However, in cases where no such model is available, it is still possible to fit an activity model using an approach based on mutual information (MI) [4, 54, 55]. This approach also requires no assumptions about the shape of the input distributions, which is particularly useful if the shape is unpredictably irregular or if the number of samples per variant is small. This is important because an incorrect assumption about either the shape of the input distributions or the mapping between activity and fluorescence could lead to biased or inconsistent results. While the use of fewer assumptions is appealing, the MI-based approach does not provide any characterization of the activity-fluorescence mapping or the properties of the input distributions, which can be biologically meaningful and of interest to the researcher.

**Fig. 4:**
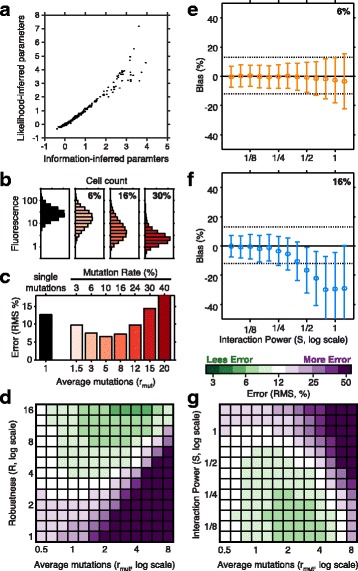
Fitting quantitative models with sort-seq data. Additive model parameters inferred from sort-seq simulations with different library designs. **a** The parameters of an additive model for the activity of a regulatory RNA [9] is inferred using either a likelihood-based or an information-based approach (*ρ*=0.99). Three values are outside the pane are poorly constrained by the information-based approach. **b** Fluorescence distributions in a targeted library (black) and random libraries characterized by different mutation rates (red). Here, simulated mutations contribute independently to activity, and the robustness is *R*=10. **c** Relative error (RMS error divided by input) in estimating the mean fluorescence for variants with a single mutation using the targeted (black) or random (red) libraries. **d** Heatmap of error as a function of the average number of mutations per variant *r*_mut_ and *R*. The color scale of the heatmap is such that the error in the targeted approach is white, and shades of purple and green indicate larger and smaller error, respectively. **e**–**f** Effect of interactions between mutations on estimates of single-mutation variants by an inferred additive model. Relative bias (circles) and standard deviation (bars) are plotted against the interaction power *S*. The dotted line indicates error of the targeted approach. **g** Heatmap of error as in **d**, plotted as a function of *r*_mut_ and *S*, for sequences with fixed *R*=10

As an example, we consider a simple additive model that is often used to quantify sequence-function relations [4, 56]. In this model (described more fully in Methods: Fitting quantitative models using sort-seq data) we assume that mutations make additive contributions to the activity of each variant. Here the task is to infer the contribution of every possible mutation in the sequence. To demonstrate the ability of sort-seq to infer these parameters, we turn to a published dataset from a sort-seq experiment that profiled the activity of a bacterial small RNA, the iron homeostasis regulator RyhB in *E. coli* [9]. In this dataset the activity of RyhB variants is characterized through the expression level of a fluorescently labeled target of repression by the small RNA. We inferred the parameters of an additive model for the activity of the small RNA using either the simple mean estimator, or an MI-based approach (see Methods: Inference of quantitative models), and found the results of the two approaches to be highly correlated (*ρ*=0.99, Fig. 4a).

When the assumptions used to define the likelihood of a model are justified, one expects the maximum likelihood approach to be as successful as maximizing MI [57]. However, the success of the simple estimator observed in Fig. 4a is not guaranteed, especially for variants close to the measurement boundaries. This success is mostly the effect of averaging over many variants, as discussed in the next section, and may not extend to parameters associated with more complex modes. For example, parameters that characterize epistatic interactions between different sequence positions are more sensitive to estimator bias, making the advantage of MLEs or MI inference more significant.

### Library design and model inference

Quantitative models predict the activity of all possible variants, including those that are not present in the library. Suppose that one is particularly interested in learning the activity of a certain variant, or a group of variants. One possible approach is to design the library in a way that guarantees that it includes these variants. We will call this the *targeted approach*. Alternatively, one can generate a library of many random variants, use them to learn a quantitative model, and use the model to predict the activity of the variants of interest, which we will call the *random approach*. This approach is likely to be simpler and less costly than the targeted one, but will it yield similar results?

To compare the two approaches we turn again to simulations. We suppose that one is interested in learning the activity of all single mutation variants of a 50 nucleotide sequence, and construct four libraries. One library (the targeted library) consists of all 150 single mutation variants (3 for each nucleotide position). To each we assign a level of activity at random, which in most cases is lower than the activity of the reference sequence. These activity values are the numbers one would like to infer. For each of the other libraries (the random libraries) we set the mutation frequency (6, 16 and 30 %), and generate 3000 random variants by introducing mutations at the appropriate frequency in the original sequence. The activity of each of the variants is determined using the additive model with same parameters as in the targeted library. Details of the simulation procedure can be found in Methods: Simulation of quantitative models. The distribution of sort-seq reads for each library, using one choice of parameters, is represented in Fig. 4b (targeted library in black, random libraries in color). With the targeted libraries, all one needs to infer is simply the mean of each variant, which we do using the simple mean estimator. With the random libraries we use the estimates from all variants (most including multiple mutations) to infer the parameters of the underlying additive model. The errors in these estimates, compared with the input parameters used in the simulation, are plotted in Fig. 4c for the same parameter set.

Surprisingly, with an equal number of reads, the estimates in the random approach can be substantially more precise than in the targeted approach. This is the case for a range of mutation frequencies that depends strongly on the resistance of the investigated system to mutations, a property of the underlying biology known as robustness and indicated by *R*. The robustness is defined as the average number of mutations required to make most variants non-functional (see Methods: Simulation of quantitative models). The random approach resulted in greater fitting power than the targeted one when *R* was larger than the average number of mutations per variant *r*_mut_ (and *R*≥2, Fig. 4d). Intuitively, in the random approach the estimate of each parameter relies on multiple sequences, whose activity is spread across a broader range of fluorescence levels, rather than on a single variant, making inference more robust. This advantage is lost however if a significant fraction of these variants are completely dysfunctional.

The random approach – unlike the targeted one – depends on having a reliable quantitative model. Naturally, one suspects that if the assumed model is wrong, the inferred parameters would be wrong as well. For example, suppose that the true relation between sequence and activity follows a model that includes pairwise interactions between bases, on top of their additive contributions, but inference is naively done using the additive model. To simulate this scenario, we construct targeted and random libraries as before, and use the true model to set the activity of each multi-mutation variant. We then use the additive model to infer the activities of single mutation variants, and calculate the RMS error in these estimates. This experiment is repeated several times, for several different activity models. Each model is characterized by the interaction power *S* as the ratio between the strength of the interaction terms and the additive terms. For larger values of *S*, there is a more substantial discrepancy between the “true” model (the one used to set the activities of multi-mutation variants) and the assumed model (the additive model used for inference).

As expected, the precision and accuracy of the estimated parameters were greatly diminished in the presence strong interactions (Fig. 4e–g). This effect was much greater for higher mutation rates. As long as *S*<1, the random approach with sufficiently low mutation rates could still result in more precise estimates than the targeted approach. Thus, a random approach, which uses a library with relatively low mutation frequency, is favorable when the assumed model provides a good (even if inaccurate) approximation to the true form of the sequence-function relation.

### The power and limitations of enrichment measurement with a single gate

An alternative approach to sort-seq is to use a single sorting gate and measure, for each variant, the ratio *ε* between the number of sorted cells (e.g. those measured above a fluorescence threshold) and the total number of cells carrying that variant [29, 30, 35–37]. Here we set aside the ambition of precise inference in favor of simplicity and considerable time and cost savings.

**Fig. 5:**
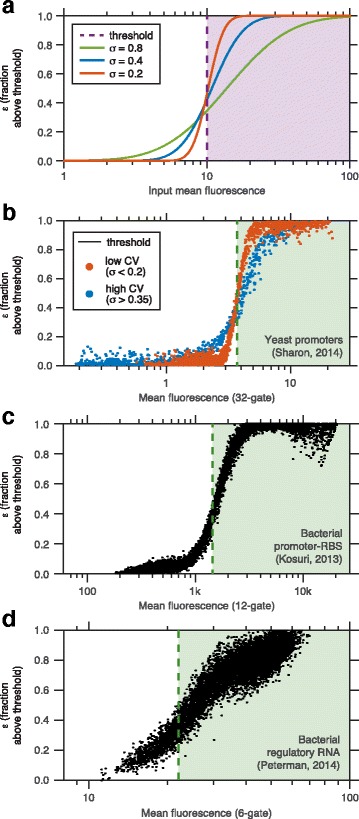
Enrichment measurements using a single sorting gate. **a** The enrichment *ε* is the the probability that a random variable takes a value larger than a given threshold. Plotted here is the enrichment for variables that follow a log normal distribution as a function of their mean, for different values of the parameter *σ*. **b**–**d** Data from three sort-seq datasets [6, 8, 9] with multiple gates. Sort-seq reads are grouped to compute the fraction of cells above a fluorescence threshold. **b** For Yeast promoter data [8], which was used to infer mean and CV, the MLE mean is compared with *ε* for different levels of CV as in Fig. 2 g. For **c** bacterial promoter-RBS [6] and **d** regulatory RNA [9] datasets, *ε* was compared to the simple mean for all variants

We reasoned that *ε* could reflect the activity of any variant whose input distribution lies both in and out of the sorting gate (Additional file 1: Figure S7). Assuming as usual log-normal input distributions, we plot the relation between the position of the input mean and the expected value of *ε* for different values of the input dispersion *σ* (Fig. 5a), showing a wider dynamical range for larger *σ*. To test this idea further, we turned to three published sort-seq datasets that used multiple gates and mimicked the enrichment approach by combining all reads from gates that exceed different thresholds as coming from the mock single gate (Fig. 5b–d, Additional file 1: Figure S8, see Methods: Reanalysis of sort-seq data). The yeast promoter study [8] and the regulatory RNA study [9] were introduced earlier. A third study synthesized and measured all pairs of roughly 100 promoters and 100 ribosome binding site (RBS) sequences, and modeled the the effect of the combination of these elements on gene expression [6].

In all cases variants for which the mean was close to the threshold showed a one-to-one relationship between the estimated mean and *ε* (Fig. 5b–d). As expected, the slope of this linear relationship depended strongly on the level of cell-to-cell variability (cf. Fig. 5b, a). In the promoter-RBS system [6], where the range of estimated means spans over two orders of magnitude and where most variants show little cell-to-cell variability, the relationship between the estimated mean and *ε* was highly non-linear, regardless of the threshold (Fig. 5c, Additional file 1: Figure S8). In contrast, the small RNA set, featuring a narrower range of mean fluorescence and greater variability for each variant [9], exhibited a clear linear relation between the two measures (Fig. 5d). In such cases a single gate configuration can be an excellent choice for tasks that do not require an accurate estimate for each variant.

**Fig. 6:**
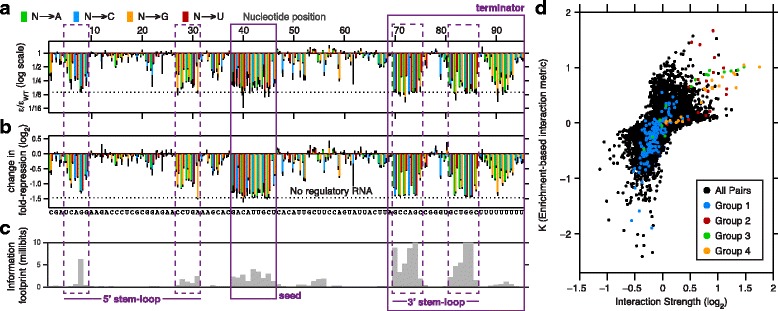
Identification of functional elements and interactions. **a**–**c** The sensitivity of each position in the regulatory RNA RyhB to mutations [9] is profiled in order to identify functional elements. Known elements are highlighted, with dashed lines indicating paired nucleotides in stem-loops of the RNA molecule. **a** Enrichment *ε* for each single-mutation variant, computed by grouping the bottom two gates. **b** Quantitative change in fold-repression, estimated by the simple mean. For both measures the value for the WT (solid line) and non-functional variants (dotted line) are indicated. **c** The information footprint, or mutual information between the nucleotide at each position and the distribution of sort-seq reads. **d** Comparison of two measures of epistasis, the inferred interaction strength (IS) and the model-free *K* based on enrichment ratios. Four groups of mutation pairs previously identified as biologically significant [9] are highlighted

An example for such an application is the identification of functional regions within the sequence of interest. In this case, one is interested in identifying regions where mutations at most positions have a significant effect on activity, but is not necessarily interested in quantifying these effects. We exemplify this application by considering the small RNA data again. Three known features of the small RNA under investigation are a 5’ stem loop required for stability, a seed sequence required for its interaction with its targets, and a 3’ stem loop required for transcription termination. In Fig. 6a–b we demonstrate that both the enrichment-based approach and quantitative inference of mean fluorescence using data from all sort bins were equally successful in identifying these functional elements and correctly defining their boundaries.

An MI-based approach, which has been introduced earlier for inferring models, can also be used for identifying functional regions in a sequence [4]. For each sequence position, one considers here the base found in a sequence read and the sorting bin in which this read was found as two random variables, and computes the MI between the two (see Methods: Reanalysis of sort-seq data). MI above background indicates that the identity of the base at that position has a significant effect on fluorescence. This approach is computationally straightforward and requires no further assumptions. In Fig. 6c we apply this method to the small RNA data and find that the identified functional elements are mostly consistent with the known functional elements, described above.

Another important use of sort-seq data is to infer epistatic interactions between functional elements [6], cellular processes [36], or single-point mutations [9, 53]. This can be done by considering a quantitative model that, in addition to the additive contributions of individual mutations, parametrizes the pairwise interactions between them. Alternatively, epistatic interactions can be investigated without a model, using single-gate assays [36]. Here, we consider a pair of mutations and compare the enrichment of variants that carry each one of them individually with the enrichment of the variant that carries both (see Methods: Measuring mutation interactions). We compared the two approaches for the regulatory RNA dataset by plotting a model-based interaction strength (IS) inferred from the full data [9], with the enrichment ratio, denoted by *K*, computed from the same data with combined gates (Fig. 6d). The two measures were well-correlated (*ρ*=0.71), indicating that the enrichment approach can offer some information about interactions.

In [9], the authors identified groups of interactions with significant biological meaning (colored dots in Fig. 6d). For example, Group 1 includes interactions between a downstream stem loop in the small RNA and its poly-U tail, which are together essential for correct termination (other groups are described in Methods: Measuring mutation interactions). Interestingly, many of these interactions stood out for both quantitatively inferred IS and with the enrichment-based *K*. However, the existence of many pairs for which the magnitude of *K* is small but IS is large, or vice-versa, suggests that *K* should not be used as a quantitative metric.

Thus, we conclude that a single-gate enrichment-based approach can be surprisingly powerful, despite its simplicity and practicality. In cases where *σ* is similar for all variants, and the log of mean fluorescence of most variants is within a range of 2*σ*, this assay can be used for applications where quantitative accuracy is dispensable. These applications include the search for highly functional sequence elements, as well as identification of potential intramolecular interactions. In the latter case, however, it is necessary to balance between low sensitivity and a high rate of false-positives.

## Conclusions

**Fig. 7:**
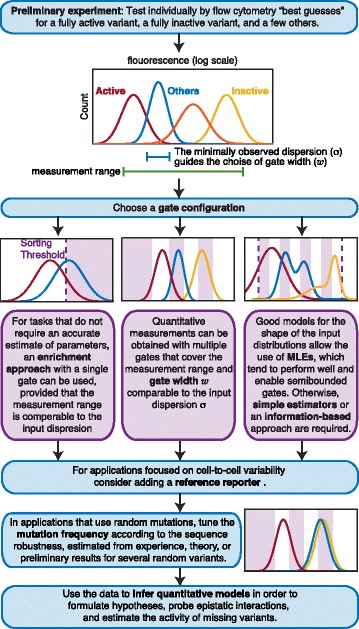
Suggested steps towards informed design of sort-seq experiments. A simple preliminary experiment has the potential to provide information about the key system parameters required for making knowledgable design choices: the measurement range, the typical dispersion of input distributions, the robustness to mutations, and the shape of the distributions required for maximum-likelihood estimators. These choices further depend on the goals of the experiment and the required resolution

Sort-seq emerges as a broad approach for studying sequence function relations across a wide range of biological processes. The massive quantitative data generated by these assays carries great promise but bears some risks. Sort-seq has the potential to provide the high-resolution data required for driving the discovery of complex and elusive phenomena, testing of quantitative models, and identification of novel molecular and functional interactions. At the same time, it is critical that we understand how design choices and analysis techniques may give rise to systematic biases [58] and lead to erroneous predictions. As described here, the use of a sound statistical approach helps to realize the full potential of the assay while mitigating these latent risks. In Fig. 7 we summarize our results by proposing a workflow that facilitates informed design choices.

The variability between cells that carry the same variant (parameterized by *σ*, the distribution width in log-scale), as compared with the overall dynamic range of the assay, has strong impact on all aspects of sort-seq. For the multiple gated approach, this scale determines the number of gates (tuned by gate width *w*) sufficient to ensure unbiased measurements. For sort-seq with a single gate, the variability determines the dynamic range around the sorting threshold. In cases where the variability differs substantially among variants, the multiple-gate approach is essential and can additionally be used to quantify this variability for each variant in the library.

In this paper we considered three inference approaches: the use of simple statistics, maximum-likelihood inference, and mutual information. Calculation of simple statistics is the simplest to implement and interpret, while maximizing mutual information involves sophisticated computation for accurate sampling, and may be more challenging for the non-expert. Both approaches require no additional assumptions, but while simple statistics provide information about the fluorescence of each variant, the MI-based approach allows direct inference of activity models. In contrast, maximum-likelihood inference requires a good model for the shape of the input distribution. When such a model is available, this approach allows simultaneous inference of activity models and noise features, and quite generally provides the largest range of estimates that are both unbiased and efficient. While maximum-likelihood inference is more computationally complex than simple estimators, standard packages are available for many popular platforms (including MATLAB, R, SciPy, and more).

Knowledge of the typical range, shape, and width of input distributions is valuable for designing the gate configuration and facilitates a maximum-likelihood approach. A useful preliminary step is therefore to characterize several variants by flow cytometry. Preferably, one should aim to explore a few variants that are spread across the range of activities, in order to define the optimal measurement range. Such variants can be generated through directed mutagenesis or de-novo DNA synthesis.

Sort-seq experiments can be used to infer parameters of quantitative models and to rigorously test alternative models. Although a sort-seq approach can yield precise measurements for a large number of variants, one can only ever expect to measure a small subset of all possible variants, since their number expands rapidly as the number of mutations increases. Model inference is therefore necessary for expounding upon the many variants that are absent from the library. When the typical number of mutations in each variant (relative e.g. to the wild-type sequence) is small, these missing variants are interpreted as complex combinations of measured variants. When this number is large, mutations carried by a missing variant of interest are probably present in many other variants in the library, which also carry other mutations. In such cases a quantitative model (such as the additive model discussed above) allows a meaningful marginalization.

## Methods

### Sort-seq simulations

In our simulated experiments, each variant was assigned an input mean *ν* and an input coefficient of variation *c*. These are the two parameters that one would like to infer. It is assumed that the fluorescence *X* in cells that carry this variant is distributed according to a log-normal distribution, whose cumulative distribution function (CDF) is given by

(1) $$\begin{array}{*{20}l} F_{\mu,\sigma}(x) = P\left(X <x |\mu,\sigma\!\right) = \frac{1}{2}\! \left(\!1+\text{erf}\left(\frac{\log x - \mu}{\sigma\sqrt{2}} \right)\right), \end{array} $$

where erf represents the error function. The parameters *μ* and *σ* of the log-normal distribution are related with the input mean *ν* and the input CV *c* through *ν*= exp(*μ*+*σ*^2^/2) and $c=\sqrt {\exp \left ({\sigma ^{2}}\right)-1}$ (and note that *c*≈*σ* for *σ*≪1).

In all simulations, the *m* sorting gates were distributed evenly on a logarithmic scale across the measurement range. Sorting gate *j* was defined by its upper and lower boundaries, *L*_*j*_ and *U*_*j*_ respectively, such that *L*_*j*+1_=*U*_*j*_ and log*U*_*j*_= log*L*_*j*_+*w*.

To simulate sort-seq we needed to compute the output distribution for each variant, that is the way in which *N* cells are distributed among the *m* sorting gates. First, the fluorescence of each cell *X* was drawn at random from the input distribution of that variant. This was done by drawing a number *y* randomly between 0 and 1, and then calculating $X = F_{\mu,\sigma }^{-1} (y)$. Next, we assigned that cell to the sorting gate that includes the value *X*, that is the gate *j* for which *L*_*j*_≤*X*<*U*_*j*_. We then counted the number *r*_*j*_ of cells that fell into gate *j*. Together we denote this list of *m* numbers by **r**. Of course, $\sum _{j=1}^{m} r_{j} = N$, which in our simulations was taken to be 100. This output distribution was then used to estimate the mean and CV, as described in the following section.

### Quantitative estimation with sort-seq

#### Simple estimators

Two approaches were used to estimate mean and CV from the output distribution **r**. The simple mean *ν*_1_ takes the population average of all sorted cells assuming each cell in gate *j* is at some fixed location within this gate, that is that its fluorescence is $\varphi _{j}=b\sqrt {L_{j} U_{j}}$, with some *b* between *e*^−*w*/2^ and *e*^*w*/2^. We chose to set *b*=*w*/(*e*^*w*/2^−*e*^−*w*/2^), which was found empirically to minimize the bias for all inputs. The simple mean is therefore

(2) $$\begin{array}{*{20}l} \hat\nu_{1} =&\ \frac1N{\sum_{j=1}^{m} r_{j} \varphi_{j}}. \end{array} $$

Similarly defined, the simple CV is

(3) $$\begin{array}{*{20}l} \hat c_{1} =&\ \frac{\sqrt{\frac1N\sum_{j=1}^{m} r_{j} \left(\varphi_{j}-\nu_{1}\right)^{2}}}{\hat \nu_{1}}. \end{array} $$

#### Maximum-likelihood estimators (MLEs)

In order to compute MLEs we first find parameters $\hat \mu $ and $\hat \sigma $ which maximize the log-likelihood function

(4) $$\begin{array}{*{20}l} \log L\left(\mu,\sigma | \textbf{r}\right) = \sum_{j=1}^{m} r_{j} \log \left(F_{\mu,\sigma}(U_{j})-F_{\mu,\sigma}(L_{j})\right). \end{array} $$

$\hat \mu $ and $\hat \sigma $ are the output of the MATLAB function fminsearch, which uses the Nelder-Mead algorithm to minimize − log*L*(*μ*,*σ*|**r**) over *μ* and *σ*, while keeping the sort-seq data **r** and the configuration parameters fixed. The MLE mean is then $\hat \nu _{2}=\exp (\hat \mu + \hat \sigma ^{2}/2)$, and the MLE CV is $\hat c_{2}=\sqrt {\exp \left (\hat \sigma ^{2}\right) - 1}$.

#### Estimator bias and efficiency

The process of simulating sort-seq reads and estimating each parameter was repeated 1000 times in order to assess the performance of these estimators. For estimator $\hat \theta $ of parameter *θ* (such as the mean or CV), bias is defined as

(5) $$\begin{array}{*{20}l} \beta_{\hat\theta} = \langle \hat\theta - \theta \rangle, \end{array} $$

where brackets indicate the average over all simulation repeats. Relative bias is accordingly $\beta _{\hat \theta }/\theta $. Precision of sort-seq estimates was quantified by MSE, $\langle (\hat \theta -\theta)^{2}\rangle $. Since estimates become more precise as *N* is increased, sort-seq estimators were compared to $\hat \theta _{0}$, the MLE of *θ* from *N* precise fluorescence measurements drawn from the same input distribution. These MLEs of mean and CV are $\hat \nu _{0} = \exp \left (\hat \mu _{0} + v_{0}/2\right)$ and $\hat c_{0} = \sqrt {\exp \left ({\hat \sigma _{0}^{2}}\right) - 1}$, where $\hat \mu _{0} = \sum _{k} \left (\log X_{k}\right)/N$ and ${\hat \sigma _{0}^{2}} = \sum _{k} \left (\log \left (X_{k}\right) - \hat \mu _{0}\right)^{2}/N$. The efficiency can then be defined as

(6) $$\begin{array}{*{20}l} \eta_{\hat\theta} = \frac{(\langle \hat\theta_{0} - \theta)^{2}\rangle}{\langle(\hat\theta - \theta)^{2}\rangle}. \end{array} $$

Because these estimators are maximally efficient for large *N*, estimators with $\eta _{\hat \theta } = 1$ are optimal. If $\hat \theta $ is an unbiased estimator based on *N* sorted cells, estimates will have similar precision to the estimator $\hat \theta _{0}$ from $N\eta _{\hat \theta }$ individually sampled fluorescence measurements.

### Reanalysis of sort-seq data

To address the effect of the input distribution on the quality of the estimates, we utilized a dataset from a previously published study [8] that aimed to infer the mean and the variance of single-cell fluorescence for each of several thousand yeast promoter variants. The published dataset (GEO accession number: GSE55346) reports the fraction of cells within each of 32 gates. Each gate *j* has an upper boundary of *U*_*j*_, a lower boundary of *L*_*j*_ and contains a fraction of all sorted cells *T*_*j*_. For each variant *i* the dataset indicates the fraction *t*_*ij*_ of its reads that appear in gate *j*, such that $\sum _{j} t_{ij} = 1$. Thus, *t*_*ij*_ is just *r*_*j*_ of the previous sections, divided by the total number of reads from this variant. This factor, however, has no bearing on the maximum-likelihood inference.

Using an assumed log-normal distribution, we inferred mean and CV for each of the 6500 promoters found in the dataset using MLEs. The gates at the negative and positive extremes were treated as censoring gates. MLEs are very sensitive to outliers, and many variants featured a small fraction of reads far from the main peak. We reasoned that the main source of these outliers is likely cells that were either mis-sorted during FACS or mis-identified during sequencing. Thus a term was added to the likelihood function corresponding to *γ*=5 *%* of all cells carrying each variant, distributed proportionally over all bins. The resulting log-likelihood is

(7) $$ \begin{aligned} \log L\left(\mu_{i},\sigma_{i} | \{t_{ij}\}\right) &= \sum_{j=1}^{m} t_{ij} \log \left(\left(1-\gamma\right) \left(F_{\mu,\sigma}(U_{j})\right.\right.\\ &\left.\left.\quad-F_{\mu,\sigma}(L_{j})\right)+ \gamma T_{j}\right). \end{aligned}  $$

Using this log-likelihood function, MLEs were computed for each variant as described in the previous section.

Parameter values estimated from the full 32-gate dataset were to be used as true parameters, thus stringent thresholding was instituted. Variants were removed based on the following criteria, which were applied in order to “replicate 1” of the published dataset: i) Variants for which there were fewer than 300 sort-seq reads corresponding to roughly 100 sorted cells (4.3 % discarded), ii) variants that had at more than 60 % of cells within a single gate, more than 90 % in two gates, or more than 40 % in either of the censoring gates (3.7 % discarded), and iii) variants for which the Kullback-Leibler divergence between the inferred distribution from the MLE and the measured distribution was greater than 0.4 bits (11.2 % discarded). This quantity was computed by comparing *t*_*ij*_ to *λ*_*ij*_, the fraction of variants in each gate as predicted by the MLE parameters $\hat \mu $ and $\hat \sigma $. These are

(8) $$\begin{array}{*{20}l} \lambda_{ij} = \left(1-\gamma\right) \left(F_{\hat\mu,\hat\sigma}(U_{j})-F_{\hat\mu,\hat\sigma}(L_{j})\right)+ \gamma T_{j}. \end{array} $$

The Kullback-Leibler divergence in bits is

(9) $$\begin{array}{*{20}l} D_{KL}\left(t_{ij} | \lambda_{ij}\right) = \sum_{j} t_{i,j} \log\left(t_{ij} / \lambda_{ij}\right). \end{array} $$

There were 5255 out of 6500 variants remaining (80.8 %) after thresholding was carried out for replicate 1. When the same procedure was performed for both replicates simultaneously, i) 10.2 %, ii) 3.7 %, and iii) 21.5 % were discarded in each step, leaving 4,202 variants remaining (64.6 %). The MLE mean and CV were well correlated between these replicates after thresholding (*r*>0.99 for the mean, *r*=0.78 for the CV, see Additional file 1: Figure S4).

Gates were re-grouped by combining sort-seq reads in adjacent gates (schematic in Fig. 2h). For example, for gates *j* and *j*+1, the newly combined gate would have boundaries *L*_*j*_ and *U*_*j*+1_, represent a fraction *T*_*j*_+*T*_*j*+1_ of all sorted cells, and contain a fraction *t*_*ij*_+*t*_*i*(*j*+1)_ of cells for each variant *i*. Regrouped configurations for replicate 1 (Figs. 2i–j and 3e–f, Additional file 1: Figures S5 and S6) had gate width *w* ranging between 0.19−0.32 for 16 gates and between 0.62−0.71 for 8 gates. Re-grouped data was used to compute the MLE mean and MLE CV using the same procedure as with the original 32 gates.

The fraction of fluorescence measurements below a threshold was estimated from three multiple-gate sort-seq datasets [6, 8, 9] using a similar re-grouping procedure. For the bacterial promoter-RBS [6] and regulatory RNA [9] datasets, we controlled for differences in the number of sequencing reads per sorted cell in each gate. Read counts from gate *j* were multiplied by the factor $d_{j}=h_{j}/\sum _{i} r_{ij}$, where *r*_*ij*_ is the number of reads corresponding to variant *i* in gate *j*, *h*_*j*_ is the total number of sorted cells from gate *j*, and the sum is over all variants identified. We then approximated the number of cells carrying variant *i* sorted by gate *j* to be *d*_*j*_*r*_*ij*_. The fraction of cells falling in gate *m*^′^ or below was therefore

(10) $$\begin{array}{*{20}l} \Phi_{i}^{m'} =&\ \frac{\sum_{j=1}^{m'} d_{j} r_{ij}}{\sum_{j=1}^{m} d_{j} r_{ij}}. \end{array} $$

For comparison, quantitative measurements of fluorescence were made using the simple mean for bacterial promoter-RBS and regulatory RNA data. This is

(11) $$\begin{array}{*{20}l} \nu_{i} = \frac{\sum_{j=1}^{m} d_{j} r_{ij} \sqrt{L_{j} U_{j}}}{\sum_{j=1}^{m} d_{j} r_{ij}}. \end{array} $$

The MLE mean was used for yeast promoter data as described above, and the fraction of cells falling in gate *m*^′^ or below was

(12) $$\begin{array}{*{20}l} \Phi_{i}^{m'} =& \sum_{j=1}^{m'} t_{ij}. \end{array} $$

For regulatory RNA data (specifically, RyhB repression of *sodB*) fold-change in target expression *f*_*i*_ for variant *i* was computed by dividing sort-seq fluorescence measurements by fluorescence measured from strains expressing non-functional variants as described previously [9], *f*_*i*_=*ν*_*i*_/*ν*_null_.

The information footprint for the regulatory RNA dataset [9] was computed as described previously [4]. Here for each nucleotide position *p*, the number of sort-seq reads in gate *j* with nucleotide *q* was computed, resulting in the table *a*_*p*_(*q,j*). This table was normalized *A*_*p*_(*q,j*)=*a*_*p*_(*q,j*)/*n*, where *n* is the total number of sort-seq reads in the experiment. The mutual information (MI) was computed between nucleotide *q* and sort-seq gate *j*, which is the information footprint

(13) $$\begin{array}{*{20}l} I_{\text{footprint}}(p) = \sum_{q,j} A_{p}(q,j) \log_{2} \left(\frac{A_{p}(q,j)}{A_{p}(q)A_{p}(j)}\right), \end{array} $$

where $A_{p}(q) = \sum _{j} A_{p}(q,j)$ and $A_{p}(j) = \sum _{q} A_{p}(q,j)$.

### Dual-reporter simulations

To address the benefits of a reference reporter, each cell in our simulations was assigned fluorescent activity *X*^(*FP*)^ for each reporter, where *FP* is the name of that reporter (typically: GFP or RFP). This value was assumed to be the product of two independent quantities, *W* which is shared among all reporters and *Z*^(*FP*)^ which is specific to each. Both *W* and *Z*^(*FP*)^ were assumed to follow a log normal distribution, which means that *X*^(*FP*)^ also followed a log-normal distribution. Moreover, if *W* has parameters *μ*_*W*_ and *σ*_*W*_ and each fluorescent reporter *Z*^(*FP*)^ has parameters *μ*(*FP*)′ and *σ*(*FP*)′, then *X*^(*FP*)^ has parameters *μ*_(*FP*)_=*μ*(*FP*)′+*μ*_*W*_ and $\sigma _{(FP)} = \sqrt {\left (\sigma _{(FP)}'\right)^{2} + {\sigma _{W}^{2}}}$. The parameters *σ*_*W*_, *σ*(*RFP*)′, and *σ*(*GFP*)′ each reflect both biological and experimental sources of variation. The distribution of *X*^(*GFP*)^/*X*^(*RFP*)^ is the same as *Z*^(*GFP*)^/*Z*^(*RFP*)^, which is log-normal with parameters *μ*_(*GFP*/*RFP*)_=*μ*(*GFP*)′−*μ*(*RFP*)′ and $\sigma _{(GFP/RFP)} = \sqrt {\left (\sigma _{(GFP)}'\right)^{2} + \left (\sigma _{(RFP)}'\right)^{2}}$. Thus when *σ*_*W*_<*σ*(*RFP*)′, the ratio GFP/RFP has a lower CV than GFP alone.

For simulations, sort-seq data was taken from the regulatory RNA sort-seq data [9] to represent a distribution of mean fluorescence and variant frequency that is typical for a sort-seq experiment. For each variant with measured mean GFP fluorescence *ν*, parameters *μ*(*GFP*)′ and *σ*(*GFP*)′ were set so that *Z*^(*GFP*)^ has mean *ν* and CV =*ν*^−1/2^. As a result $\sigma _{(GFP)}' = \sqrt {\log \left (1 + \nu ^{-1}\right)}$ and $\mu _{(GFP)}' = \log \left (\nu /\sqrt {1+\nu ^{-1}}\right)$. Distributions for *W* and *RFP* were assumed to remain fixed with *σ*_*W*_=0.40 and *σ*(*RFP*)′=0.15. The parameters *μ*_*W*_ and *μ*(*RFP*)′ contribute only a constant scaling factor to all measurements, making their choice irrelevant for any of the computed results. In our simulations we simply set them to zero.

Using these input parameters for each variant, sort-seq simulations were carried out as in Methods: Sort-seq simulations. For each variant, $\sum _{j} d_{j} r_{ij}$ sorted cells were used. Input parameters *μ*_(*GFP*)_ and *σ*_(*GFP*)_ were used for the single reporter, whereas *μ*_(*GFP*/*RFP*)_ and *σ*_(*GFP*/*RFP*)_ were used for the dual reporter. A cautious gate configuration (*w*=0.15, covering more than 2 orders of magnitude) was used. Simple estimators were used to estimate the mean and CV for each variant, as in “Methods: Quantitative estimation with sort-seq”.

### Inference of quantitative models

Two approaches were used to fit sort-seq data from a previously published sort-seq dataset [9] to an additive model. For each variant, an “activity” function *G* was determined from **Q**^(*i*)^, the sequence vector for variant *i* which is composed of nucleotides $Q_{p}^{(i)}$ (= A, C, G, or T) at each position *p* (=1,2,…94, the number of nucleotides in the sequence under study). In the additive model

(14) $$\begin{array}{*{20}l} G \left(\mathbf{Q}^{(i)}\right) = G_{0} + \sum_{p,q} H_{p,q} I\left(q=Q_{p}^{(i)}\right), \end{array} $$

where *I*(*B*) is the indicator function that is 1 if the statement *B* is true and 0 otherwise, and *G*_0_ and *H*_*p,q*_ are the parameters of the model that need to be inferred.

In the first approach, sort-seq reads were used to estimate mean fluorescence for each variant $\hat \nu _{i}$ using the simple mean as in Methods: Reanalysis of sort-seq data. The mapping between activity and measured fluorescence was done using a two-state model that is frequently used in modeling regulatory systems, such that $\nu _{i} = \nu _{\text {null}} \left (1+e^{G \left (\mathbf {Q}^{(i)}\right) }\right)^{-1}$. Here again *ν*_null_ is the mean fluorescence in cells that carry no small RNA. Model parameters were then optimized to minimize mean squared difference between the model prediction and the estimates of $\hat \nu _{i}$ using the MATLAB function lsqnonlin.

In the second approach, we look for parameters that would maximize the mutual information between the model *G* and the sort-seq measurements. The approach was implemented following earlier work which characterized a bacterial promoter [4] (recently reviewed in [54]). We begin by organizing the sort-seq data in a binary table *D*_*ij*_. The *i*th row in the table corresponds to read *i* and we let *D*_*ij*_=1 if the read was found in sorting gate *j* and 0 otherwise.

With a given set of parameters **H** of the model *G*, we compute *G*_*i*_(**H**) for each row of the table according to the sequence of the corresponding read. We let *D*(**H**) be table *D* with its rows sorted in ascending order of *G*_*i*_(**H**). Since *D*(**H**) is a very large table and difficult to handle, we reduce the number of rows by combining blocks of 1000 rows. The compressed matrix is then multiplied on the left by a gaussian filter

(15) $$ \begin{aligned} \Lambda_{ij} &= \frac{1}{2} \text{erf}\left(\frac{i-j}{n\sqrt{2} / (100 \times 1000)}\right) \\&\quad-\frac{1}{2}\text{erf}\left(\frac{i-j-1}{n\sqrt{2} / (100 \times 1000)}\right), \end{aligned}  $$

where erf is the error function and *n* is the total number of sort-seq reads. The resulting table $\tilde D(\textbf {H})$ can be thought of as a joint distribution between the rank of each row and the distribution of the reads it represents across the sorting gates. Appropriately, the sum of its elements is 1. From this table, we compute the mutual information

(16) $$\begin{array}{*{20}l} MI(\textbf{H}) = \sum_{i,j} \tilde D_{ij}(\textbf{H}) \log_{2} \left(\frac{\tilde D_{ij}(\textbf{H})} {\tilde D_{i}(\textbf{H}) \tilde D_{j}(\textbf{H})}\right), \end{array} $$

where $\tilde D_{i}(\textbf {H}) = \sum _{j} \tilde D_{ij}(\textbf {H})$ and $\tilde D_{j}(\textbf {H}) = \sum _{i} \tilde D_{ij}(\textbf {H})$.

We use a Monte Carlo approach to find the set of parameters **H** which maximized *MI*(**H**) [4]. First, one of the parameters *H*_*p,q*_ chosen randomly and perturbed by adding a normally distributed random variable with mean 0 and variance 1. Parameters were re-normalized after each step as a proportional increase of all parameters does not affect the ranking of *G*. Second, *MI* was computed using the adjusted parameters. Third, it was decided whether to keep or reverse the parameter change. If the mutual information remained fixed or increased this change was kept. If *MI* was decreased by *δ*, the parameter change was reversed with probability 1−2^−*nδ*^. This was achieved by drawing a random number between *x* between 0 and 1. If *x*<2^−*nδ*^ the change was kept; otherwise it was reversed. These steps were iterated until parameters and mutual information converged (>300,000 steps).

The results did not depend on the initial choice of parameters. Previous work also included a temperature exchange procedure in order to avoid getting trapped at local maxima of the mutual information in parameter space [4]. In our analysis with the regulatory RNA dataset this did not occur and we achieved similar results with or without this additional step. Model parameters were presented as ensemble averages over continued iteration of the Monte Carlo procedure.

### Simulation of quantitative models

To test the implications of a discrepancy between the true sequence-function mapping and the assumed model, we explored two cases: one where the activity of the variants follow an additive model, and one where these activities follow a model that includes interactions between bases at different positions. To construct the random libraries, we first determined the sequences of 3000 participating variants. Each library is defined by *r*_mut_, the average number of mutations per variant compared with the reference sequence, which was taken arbitrarily to be a sequence of 50 ‘A’s. The sequence of each variant in the library was determined by deciding for each position if it takes the reference value ‘A’ (with probablity 1−*r*_mut_/50)) or not. In the latter case, a base ‘G’, ‘C’, or ‘T’ was assigned at random. In addition we constructed the targeted library, which consisted of the 50-‘A’ reference sequence and all other 150 single-position mutants.

Next, we determined the activity associated with each variant. In the additive case, we used the model described in the previous section, with *G*_0_=3.0 and parameters for *H*_*p,q*_ that were drawn from a normal distribution with mean −*G*_0_/*R* and standard deviation 4*G*_0_/3*R*. Here *R* is robustness, the number of mutations for which *G*<0 for 50 % of variants. These numbers were selected with the MATLAB function normrnd.

In the interacting model, the activity *G*_*I*_ was defined as

(17) $${} {\fontsize{8.9pt}{8.9pt}{\begin{aligned} G_{I}&\left(\mathbf{Q}^{(i)}\right) = G+\sum_{p_{1},q_{1},p_{2},q_{2}}\!\! J_{p_{1},q_{1},p_{2},q_{2}} I\left(q_{1}=Q_{p_{1}}^{(i)}\right) I\left(q_{2}=Q_{p_{2}}^{(i)}\right). \end{aligned}}}  $$

Here *G* is the activity of the additive mode, with the parameters *G*_0_ and *H*_*p,q*_ selected as before. The matrix $J_{p_{1},q_{1},p_{2},q_{2}}$ accounts for the interactions between pairs of bases. Its parameters were chosen from a normal distribution with mean 0 and standard deviation *SG*_0_/*R*, where *S* is the Interaction Power.

Finally, we generated the sort-seq data for each library. For each random library, we picked a single cell from each variant, and for the targeted library we picked 20 cells per variant. The fluorescence level of these cells was taken from the input distribution, whose CV was 0.5 and mean *ν*_*i*_ was determined by the activity of each variant,

(18) $$\begin{array}{*{20}l} \nu_{i} = 1 + e^{G\left(\mathbf{Q}^{(i)} \right)}. \end{array} $$

We then determined which of 24 sorting gates (with width *w*=0.3) contained the fluorescence value. This multiple-gate configuration was used in order to avoid contributing bias to the measurement. Simulated sort-seq data from each library of variants was used to fit the additive model for *ν* using the first approach in Methods: Inference of quantitative models. MATLAB code for these simulations is available upon request.

### Measuring mutation interactions

The method for identifying interactions by computing Interaction Strength (*IS*) was derived and discussed previously [9]. Briefly, by modeling fold-change measurements *f*_*i*_ with the additive model described above, fold-change for variants with 2 or more mutations could be predicted using data from the wild-type and variants with a single mutation, which were used to fix the relevant parameters of the model *G*_0_ and *H*_*p,q*_. As a result, given fold change measurements for the wild-type (*WT*) and two variants each with a single mutation, *α* and *β*, the predicted fold-change for the double mutant (for *f*_*αβ*_) is

(19) $$\begin{array}{*{20}l} f_{\text{pred}} = \frac{f_{WT}^{-1} - 1}{f_{WT}^{-1} - 1 + \left(\,f_{\alpha}^{-1} - 1\right)\left(\,f_{\beta}^{-1} - 1\right)}\;. \end{array} $$

*IS* for the pair of mutations *α* and *β* is defined as *f*_pred_/*f*_*αβ*_. Using *ε*_*i*_, the the fraction of sort-seq reads in the bottom two gates, the enrichment ratio used to probe interactions is

(20) $$\begin{array}{*{20}l} K_{\alpha\beta}= \log\left(\frac{\varepsilon_{\alpha\beta} \varepsilon_{\text{WT}}}{\varepsilon_{\alpha}\varepsilon_{\beta}}\right). \end{array} $$

Groups of mutations in the small RNA RyhB of particular biological significance were identified in [9] and highlighted in Fig. 6d. Group 1 includes interactions between mutations in a downstream stem loop and mutations with the poly-U tail of the RNA molecule, which together ensure correct termination of the its transcription. Group 2 includes pairs of compensatory mutations that individually break but together restore Watson-Crick base pairing in double-stranded parts of the molecule. Groups 3 and 4 include pairs of mutations, one of which is found in the region of the small RNA that binds its targets, and the other is found in separate structures (A30G in Group 3 and U55A in Group 4). These two groups of interactions demonstrate a balance between specificity and efficiency in small RNA regulation.

### Ethics statement

No human or animal data was used in this study, therefore no ethics statement is necessary.

## Additional file

Additional file 1 Supplementary Figures with figure legends. Figure S1: Bias and efficiency of the simple mean estimator. Figure S2: Bias and efficiency of maximum likelihood estimators. Figure S3: Bias and efficiency of the simple CV estimator. Figure S4: MLE mean and CV using a yeast-promoter dataset. Figure S5: MLE mean for sort-seq data with combined gates. Figure S6: MLE CV for sort-seq data with combined gates. Figure S7: Enrichment measurements from different input distributions. Figure S8: Enrichment estimates using re-grouped gates from two sort-seq datasets. (PDF 3755 kb)

## Abbreviations

CV: coefficient of variation
FACS: fluorescence activated cell sorting
GFP: green fluorescent protein
IS: interaction strength
MI: mutual Information
MLE: maximum likelihood estimator
MSE: mean squared error
PWM: position weight matrix
RFP: red fluorescent protein
RMS: root mean squared
WT: wild-type
