# Supplementary material for: Sort-seq under the hood: implications of design choices on large-scale characterization of sequence-function relations
Source: BMC Genomics. 2016 Mar 9;17:206. doi: 10.1186/s12864-016-2533-5 (PMC4784318; doi:10.1186/s12864-016-2533-5)
Supplement: Additional file 1 — Supplementary Figures with figure legends. Figure S1: Bias and efficiency of the simple mean estimator. Figure S2: Bias and efficiency of maximum likelihood estimators. Figure S3: Bias and efficiency of the simple CV estimator. Figure S4: MLE mean and CV using a yeast-promoter dataset. Figure S5: MLE mean for sort-seq data with combined gates. Figure S6: MLE CV for sort-seq data with combined gates. Figure S7: Enrichment measurements from different input distributions. Figure S8: Enrichment estimates using re-grouped gates from two sort-seq datasets. (PDF 3755 kb) [file 12864_2016_2533_MOESM1_ESM.pdf]

Supplementary Figures

Sort-seq under the hood: implications of design choices on large-scale characterization of sequence-function relations

Neil Peterman[1] and Erel Levine[1*]
1. Harvard University, Dept. of Physics and FAS Center for Systems Biology, 17 Oxford St., Cambridge MA, USA
* Corresponding author: Erel Levine, elevine@fas.harvard.edu

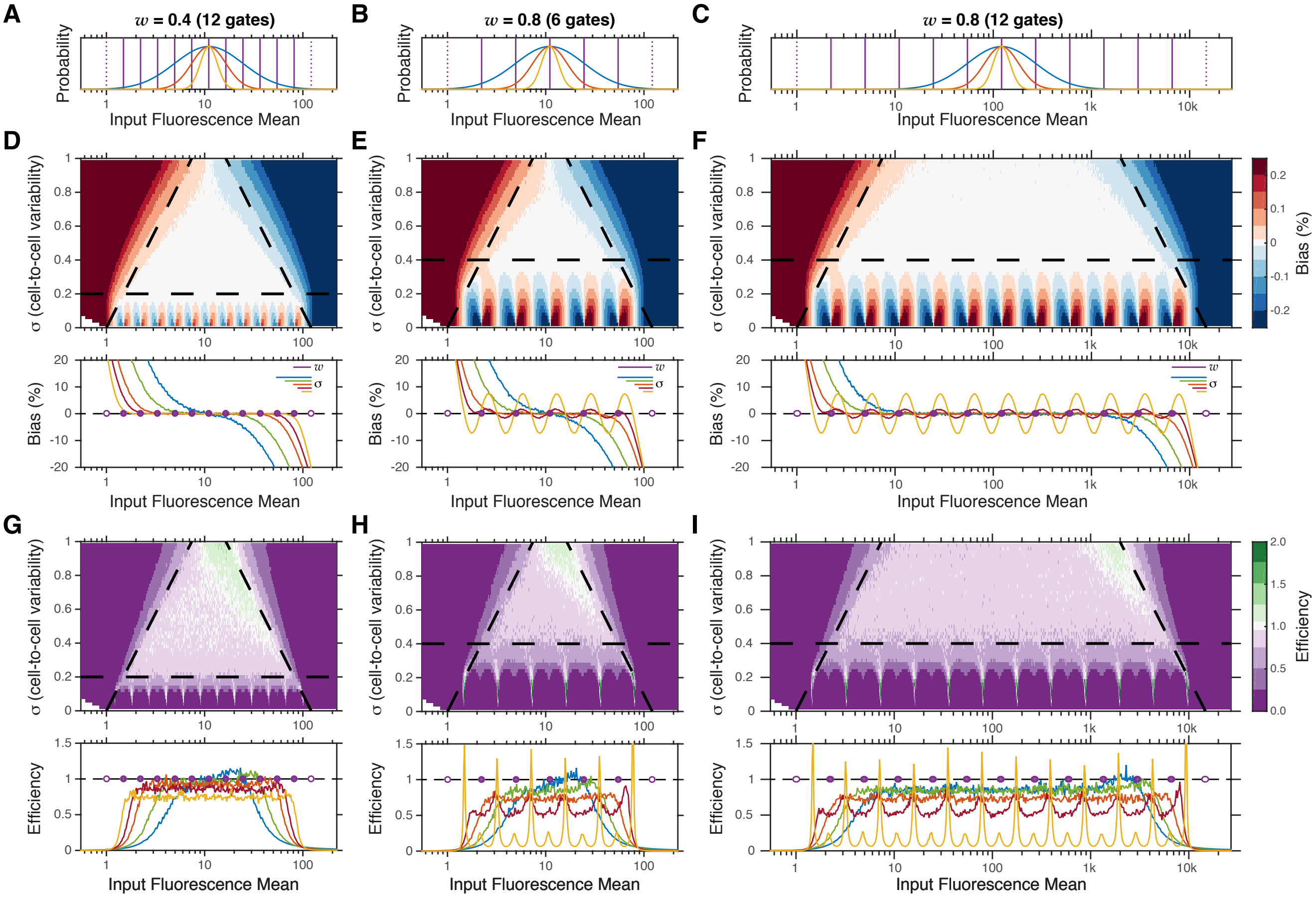

Supplemental Figure S1: Bias and efficiency of the simple mean estimator. (A-C) Three configurations of log-spaced sort gates, presented as in Fig. 2A. (A) 12 gates and $w = 0.4$, (B) 6 gates and $w = 0.8$ as in Figs. 2-3, and (C) 12 gates and $w = 0.8$. (D-F) Heatmap of relative bias for the simple mean estimator as a function of input mean (ν) and σ for each configuration. White regions represent negligible bias (within 1% of the input). Dashed black lines bound the region where the simple mean has low bias, $\sigma > w/2$, $\nu > \ell e^{2\sigma}$ and $\nu < ue^{-2\sigma}$. Below are plots of relative bias for several levels of variability as in Fig. 2B. Purple circles indicate gate boundaries, with open circles corresponding to measurement boundaries. (G-I) Heatmap of efficiency for each configuration. White regions indicate efficiency between 0.95-1.05. Below are plots of efficiency as in Fig. 2C. For all simulations $N = 100$ sorted cells per repeat were used.

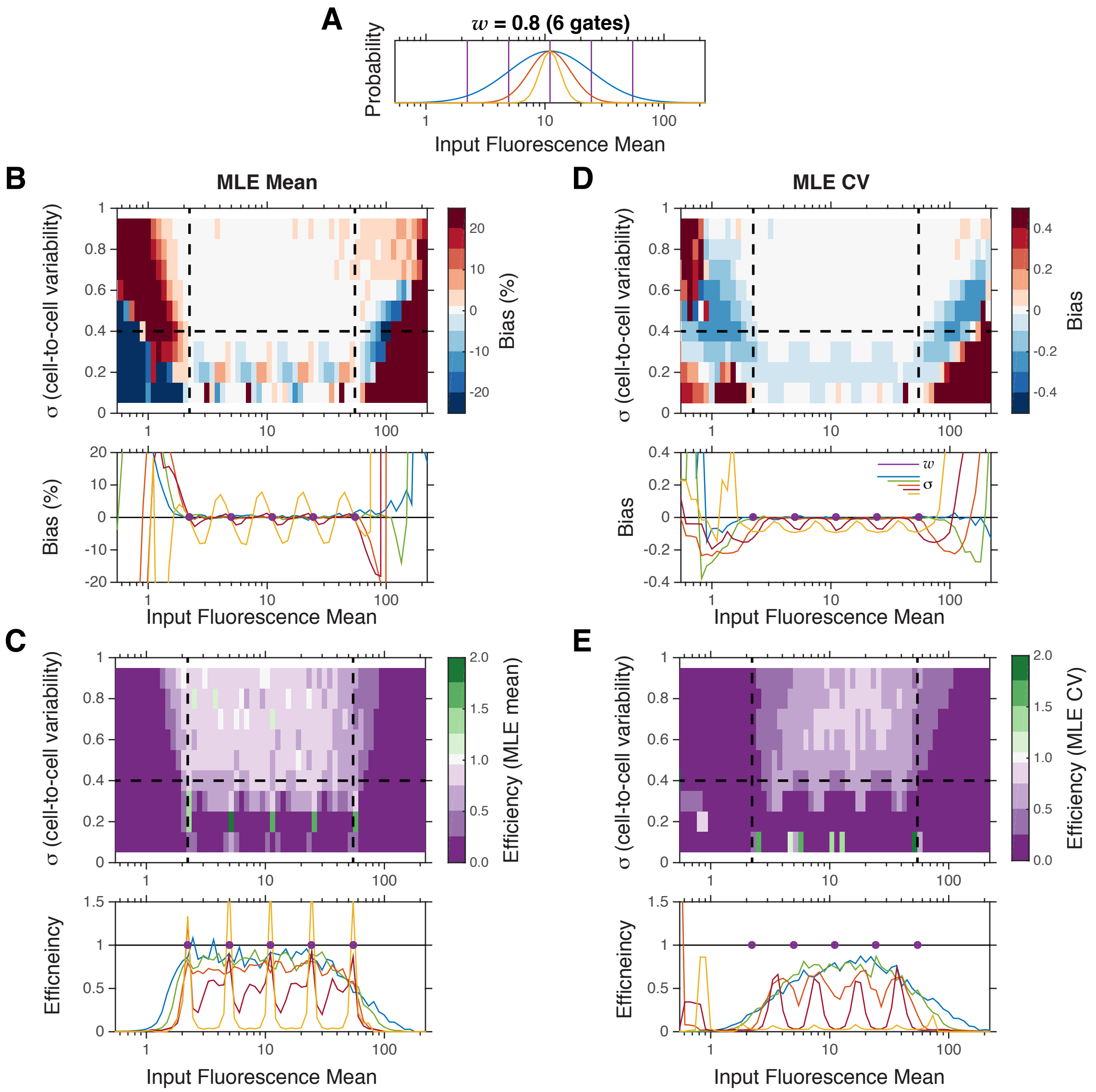

Supplementary Figure S2: Bias and efficiency of maximum likelihood estimators. Bias and efficiency of the MLE mean and CV using a configuration with 6 gates (as in Figs. 2-3), plotted as in Supp. Fig. S1. (A) Sorting configuration with 6 gates, including two semibound gates at the extremes and 4 log-spaced gates ($w = 0.8$). (B) Relative bias and (C) efficiency of the MLE mean, as plotted in Supp. Fig. S1. Dashed black lines bound the region with negligible bias, here $\sigma > w/2$, $\nu > \ell + w$ and $\nu < u - w$. $\ell + w$ and $u - w$ are respectively the upper and lower boundaries of the two semibound gates. (D) Bias and (E) efficiency plotted similarly for the MLE CV. For the CV, white regions on the heatmaps indicate absolute bias is less than 0.02 or efficiency is between 0.95-1.05.

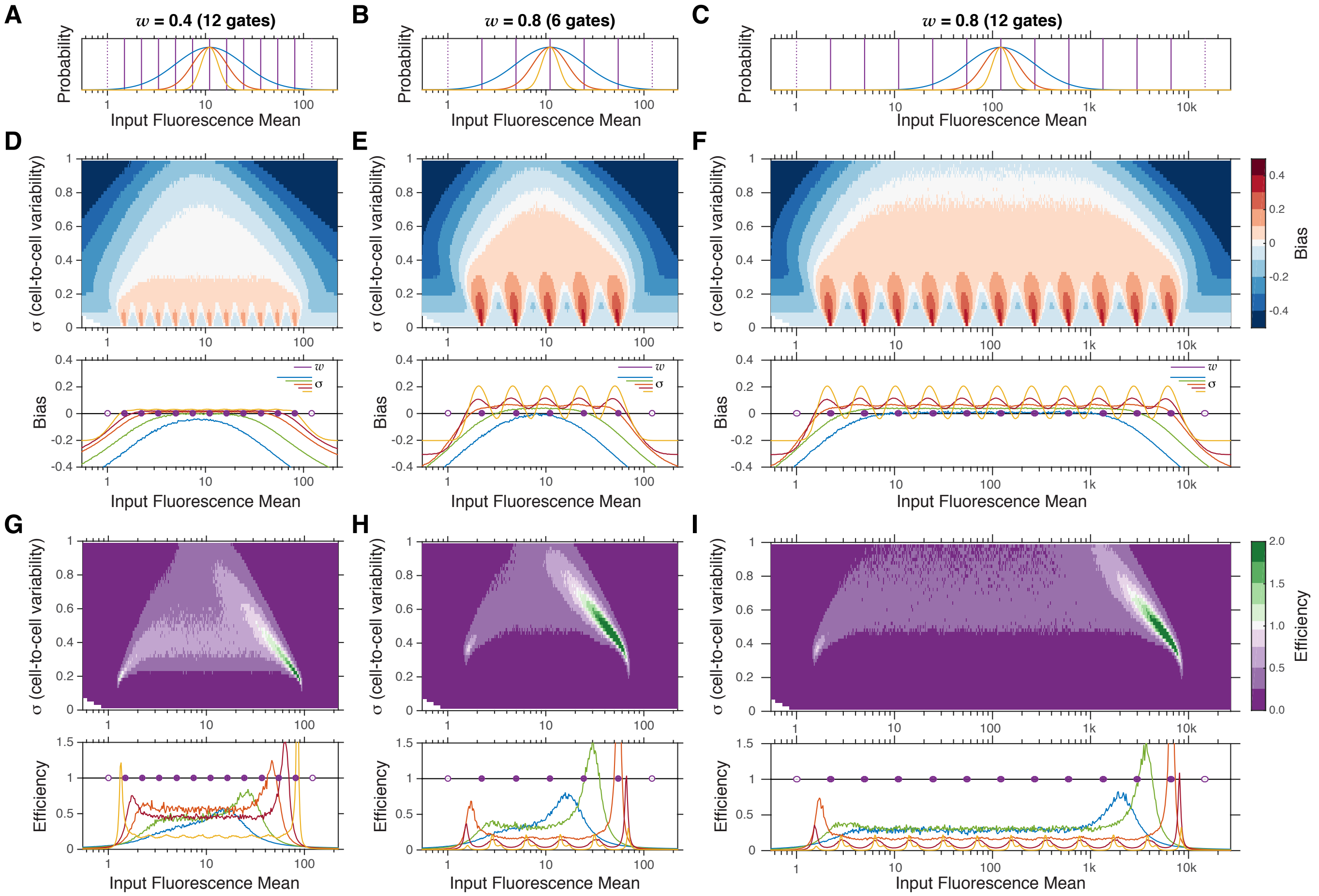

Supplementary Figure S3: Bias and efficiency of the simple CV estimator. (A-C) The same sorting configurations as in Supp. Fig. S1. (D-F) Bias of the simple CV estimator for each of the configurations, plotted as in Supp. FIg. S2D. Below are plots as in Fig. 3A. (G-I) Efficiency of the simple CV estimator for each configuration, plotted as in Supp. FIg. S2E.

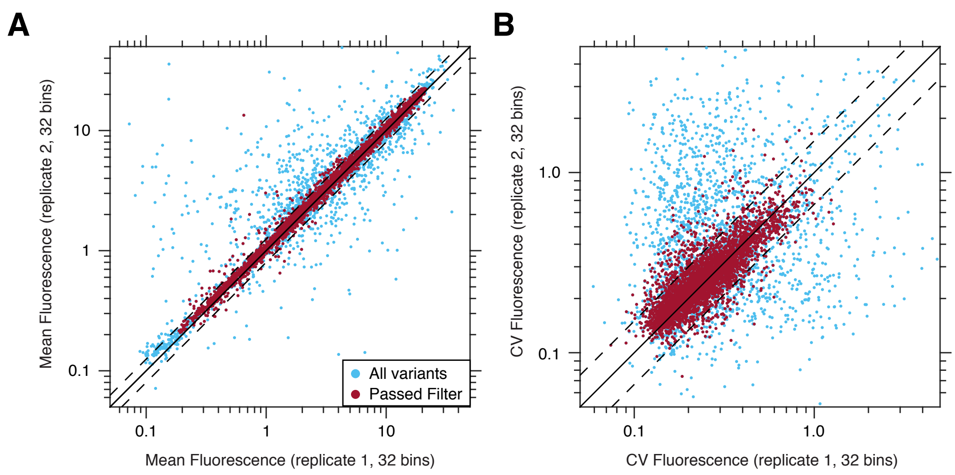

Supplementary Figure S4: MLE mean and CV using a yeast-promoter dataset. Mean and CV were inferred from the yeast promoter dataset [8] using MLEs and an assumed log-normal distribution. Data included two biological replicates for 6,500 variants. After filtering data from each replicate separately (see Methods: Reanalysis of sort-seq data), 4,202 (64.6%) variants remained. (A) Inferred MLE mean was compared between replicates. Estimates were highly correlated between replicates for filtered variants (Pearson's correlation r=0.996), with 97.1% within 1.25-fold of their replicate (dashed lines). (B) MLE CV was compared similarly. CV estimates were also correlated for filtered variants (r=0.781), with 91.4% within a factor of 1.5-fold of their replicate (dashed lines).

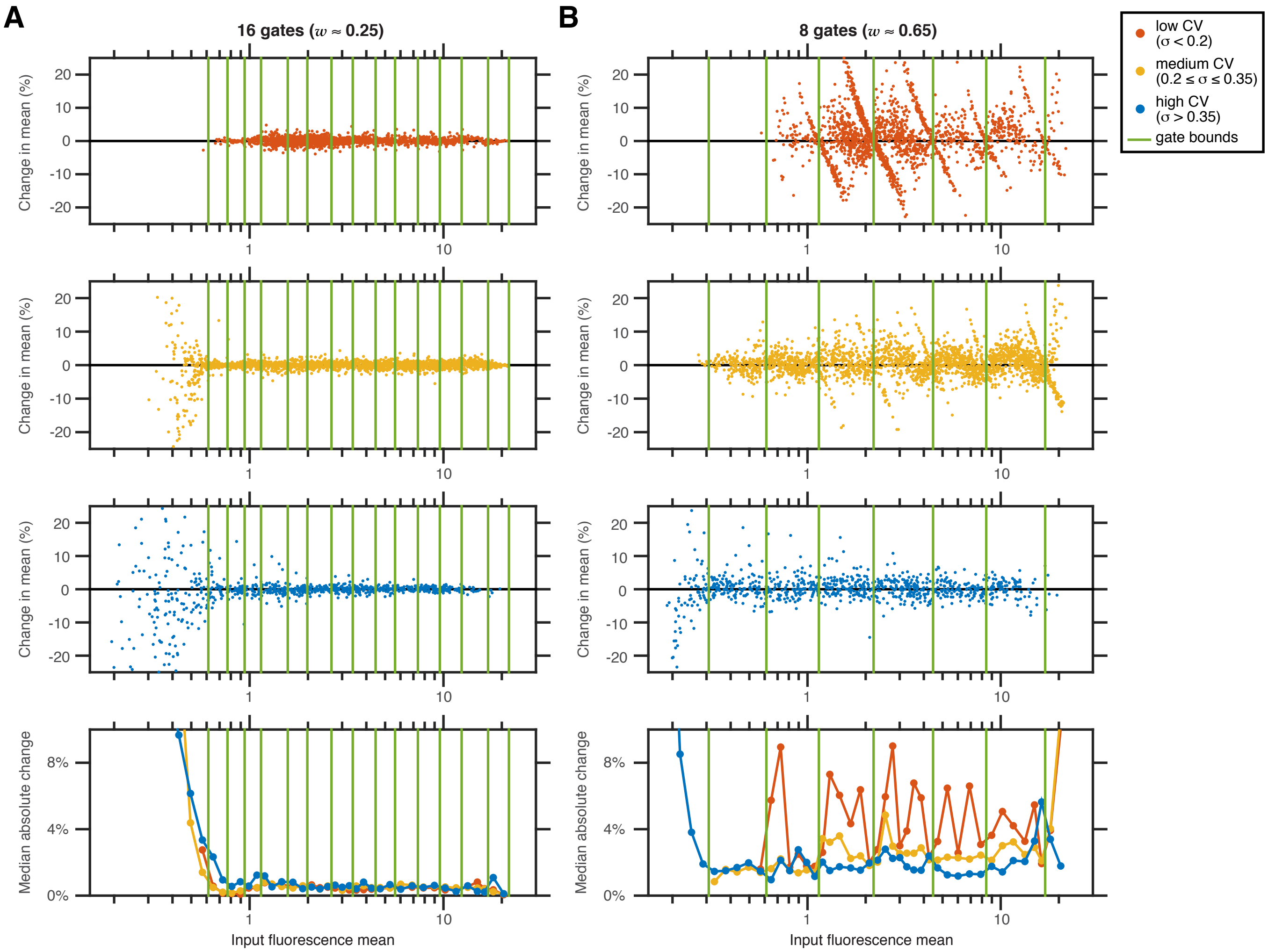

Supplementary Figure S5: MLE mean for sort-seq data with combined gates. Data as in Fig. 2I-J. Mean was inferred using combined gate configurations with (A) 16 gates and (B) 8 gates. Relative changes between the estimates of the mean using the original data (32 gates) and re-grouped data, plotted separately for variants with low cell-to-cell variability ($\sigma < 0.2$, red, 2,044 variants), medium variability ($0.2 \leq \sigma \geq 0.35$, amber, 2,338 variants), and high variability ($\sigma > 0.35$, blue, 873 variants).

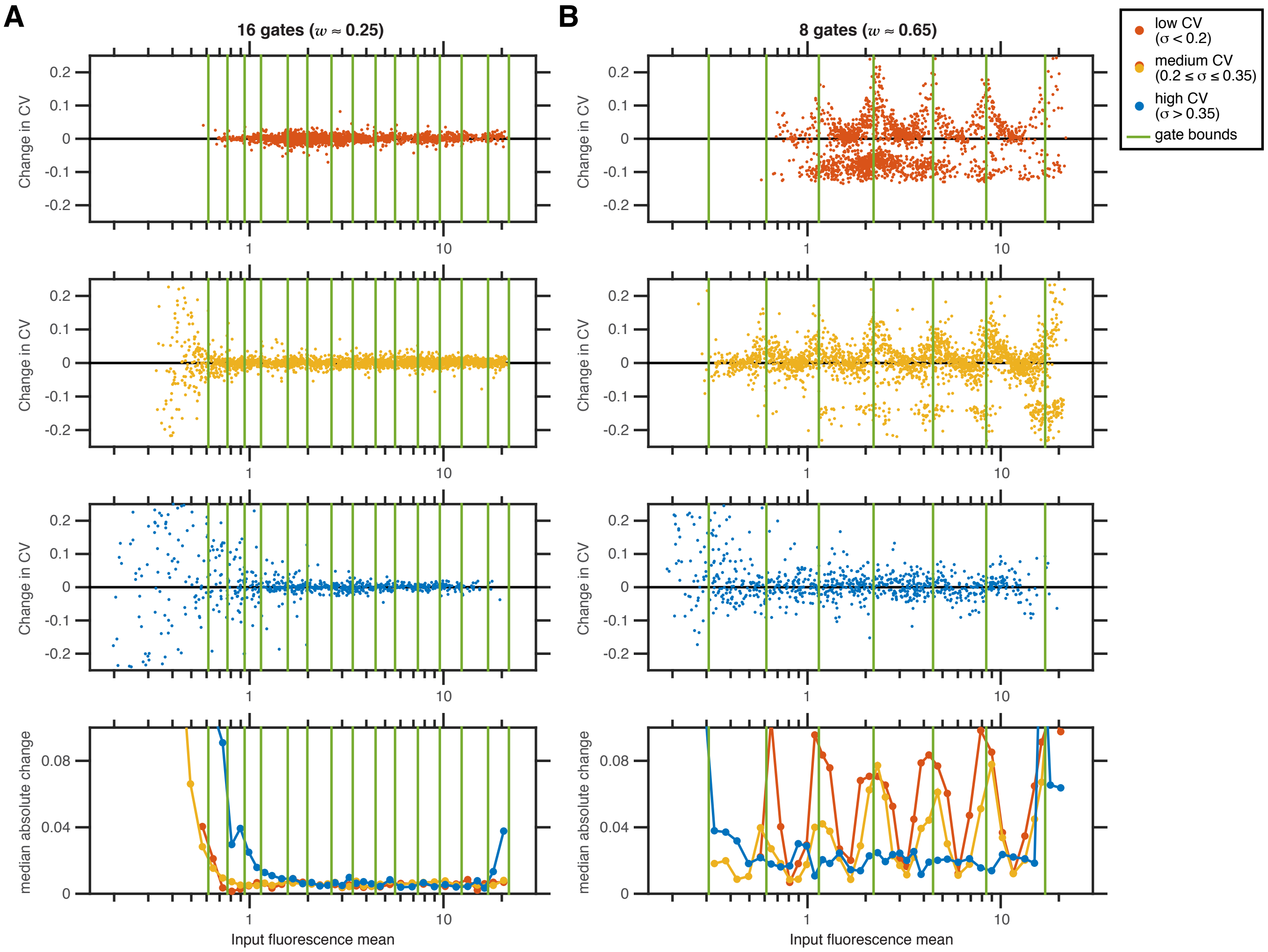

Supplementary Figure S6: MLE CV for sort-seq data with combined gates. Data as in Fig. 3E-F. CV was inferred using combined gate configurations with (A) 16 gates and (B) 8 gates. Changes in CV estimates using the original data (32 gates) and re-grouped data are plotted separately for variants with different levels of cell-to-cell variability, as in Supp. Fig. S5.

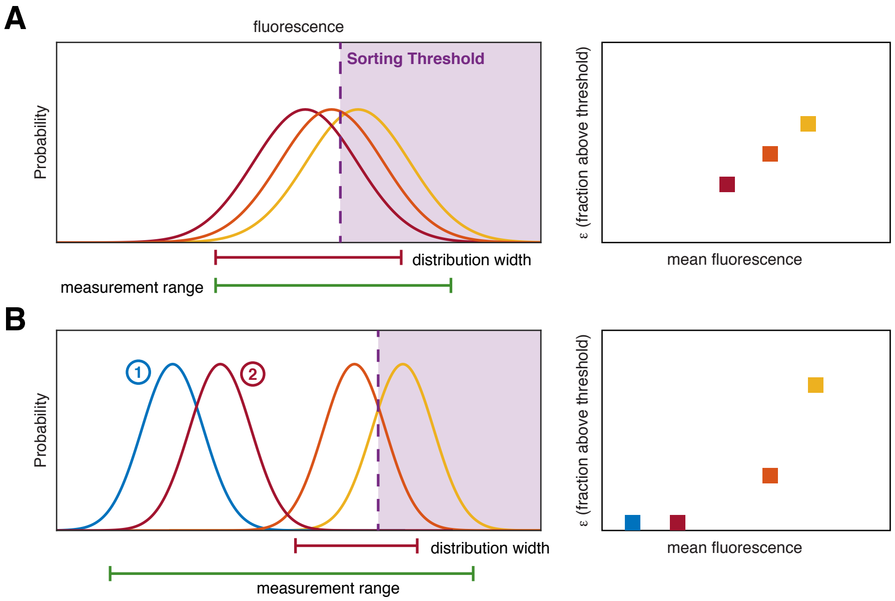

Supplementary Figure S7: Enrichment measurements from different input distributions. Fluorescence histograms for several variants. Panels on the right relate input mean fluorescence and the fraction above the threshold, similar to Fig. 5A. (A) When the distribution width is similar to the measurement range, the fraction of cells above the threshold reflects increases in mean fluorescence. (B) When the distribution width is much smaller than the measurement range, for any choice of threshold some variants will have only a small fraction of cells sorted or unsorted (here, variants 1 and 2). The substantial fold difference between variants 1 and 2 here does not have any measurable effect on enrichment.

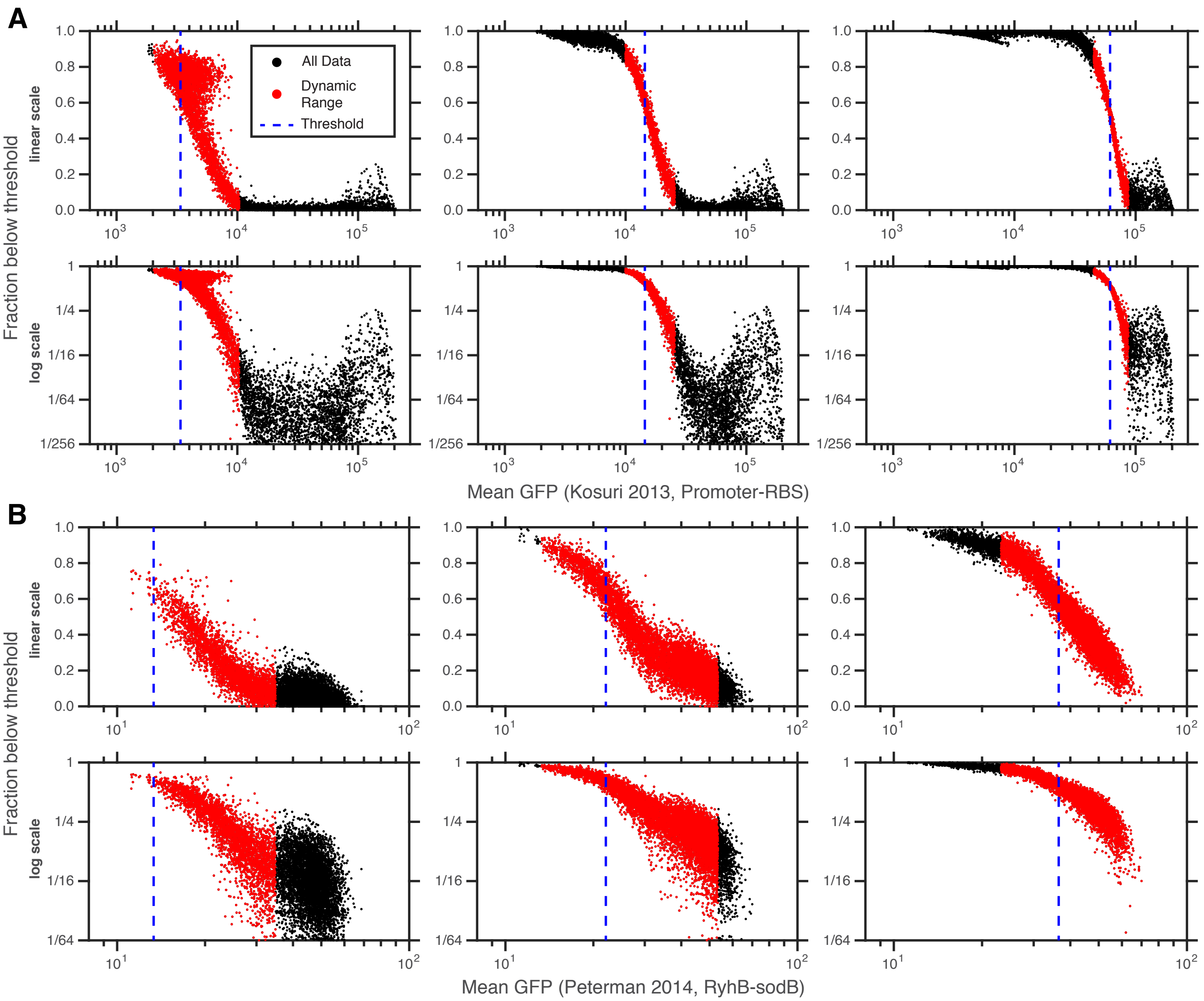

Supplementary Figure S8: Enrichment estimates using re-grouped gates from two sort-seq datasets. Data as in Fig. 4C-D with three different fluorescence thresholds. (A) Promoter-RBS data [6], in which sort-seq was performed with 12 gates over more than 2 orders of magnitude of fluorescence activity. (B) Regulatory RNA data [9] (RyhB repression of *sodB*), for which 6 gates were used over approximately 1 order of magnitude. Variants within the dynamic range are indicated in red. For the RBS-promoter data, these fractions of variants within this range for the low, medium and high thresholds were respectively 43%, 18% and 20%. For regulatory RNA data these fractions were respectively 33%, 91% and 87%.
